# A New Method for In-Situ Skin Penetration Analysis by Confocal Raman Microscopy

**DOI:** 10.3390/molecules25184222

**Published:** 2020-09-15

**Authors:** Richard Krombholz, Dominique Lunter

**Affiliations:** Department of Pharmaceutical Technology, Eberhard Karls University, Auf der Morgenstelle 8, 72076 Tuebingen, Germany; richard-paul.krombholz@uni-tuebingen.de

**Keywords:** confocal Raman spectroscopy (CRM), skin penetration, depth profiling

## Abstract

In the development of dermal drug formulations and cosmetics, understanding the penetration properties of the active ingredients is crucial. Given that widespread methods, including tape stripping, lack in spatial resolution, while being time- and labour-intensive, Confocal Raman Microscopy is a promising alternative. In optimizing topically applied formulations, or the development of generic formulations, comparative in-situ measurements have a huge potential of saving time and resources. In this work, we show our approach to in-situ skin penetration analysis by confocal Raman Microscopy. To analyse feasibility of the approach, we used caffeine solutions as model vehicles and tested the effectiveness of 1,2-pentanediol as a penetration enhancer for delivery to the skin.

## 1. Introduction

The skin is the largest organ in the body and its functions are varied. Its provides an external barrier, protecting the body from external damage or water loss, as well as controls the body temperature and plays an important role in metabolism. Understanding how the barrier function works and in which rate and extend active ingredients penetrate the skin is extremely important [1,2] when it comes to topically applied drug dosage forms and cosmetics. The outermost layer, the stratum corneum (SC), provides this barrier function [3]. It is composed of keratin-filled corneocytes, embedded in a lipid-matrix and reaches a thickness of 15–20 µm [4]. The skin plays an important role in the penetration of topically applied substances because of its main function as a barrier.

To understand drug delivery of topically applied dosage forms, it is important to understand the rate and extent of the active ingredient penetrating into the SC, especially when it comes to optimizing formulations [5,6]. The number of analytical methods for measuring penetration properties of drugs are limited. Most commonly, the skin is incubated with the formulation to be tested, and segmented afterwards, either by removing the SC step by step with adhesive tape strips, or by cutting it using a cryo-microtome [7,8,9]. The amount of drugs in each layer can be determined by High-performance liquid chromatography (HPLC) subsequently. This procedure is very time consuming and labour intensive because every measuring point has to be incubated separately, due to the subsequent destruction of the skin sample. Optical methods have the advantage of being non-destructive, which allows continuous measurements [10]. Confocal Raman microscopy (CRM) has been described as a promising technique, especially because it does not rely on labels or dyes. For measuring deeper penetrations, spatially offset Raman spectroscopy (SORS) is described as an effective method to obtain stronger signals in even deeper sample layers, than in this study [11,12]. Direct drug quantification is possible because of the linear correlation of molecule concentration and Raman scattering [13]. So far, CRM has been used in many studies to acquire penetration profiles of several drugs in vivo and in vitro [14,15,16,17].

We focused on ex-vivo-experiments as in-vivo measurements face ethical difficulties and are not possible in early stages of formulation development. Most of the published studies describe a separate incubation step or a cross-sectioning of the skin before CRM measurements, while in-situ-measurements are still uncommon. In-situ measurements have a huge advantage of reducing the measuring time to the time of incubation, while providing real-time information. This means the amount of measuring points can also be increased.

The aim of this study was to develop a penetration cell, which enables the in situ measurement of drug penetration into the skin, in order to promote CRM as a standard analytical technique for drug depth profiling. Caffeine was used as a hydrophilic model drug, as it is commonly used in skin penetration as a reference drug [18]. To show the effectiveness of the model, a solution of caffeine in water was compared to a solution containing 1,2-pentanediol as a penetration enhancer.

## 2. Materials and Methods

### 2.1. Materials

Caffeine was obtained from Caesar & Loretz GmbH (Hilden, Germany). 1,2-Pentandiol was provided by BASF SE (Ludwigshafen, Germany), Parafilm was purchased from Bemis Company Inc. (Oshkosh, WI, USA). Sodium chloride, potassium chloride, disodium phosphate and monopotassium phosphate, as used for the phosphate buffered saline (PBS) pH 7.4 were all of European Pharmacopeia Grade. For the preparation of all solution ultra-pure water (Elga Maxima, High Wycombe, UK) was used. Porcine ear skins (German land race) were provided by a local butcher.

### 2.2. Preparation of Porcine Ear Skin

For the penetration experiments porcine ear skin was used because of its similarity regarding histology and morphology to human skin [19,20,21].

All ears used for the experiments were provided by a local butcher (Bio Metzgerei Griesshaber, Moessingen-Oeschingen, Germany). The porcine ears were picked up right after the animals were butchered. The Department of Pharmaceutical Technology is registered for the use of animal products at the District Office of Tuebingen (registration number: DE 08 416 1052 21) [22]. The fresh porcine ears were cleaned with isotonic saline and cotton swabs. Afterwards, the full-thickness skin was cut off the cartilage and, where necessary, cleaned again using cotton swabs and isotonic saline. The skin was cut into about 4 cm wide strips and fixed with pins onto an aluminium foil wrapped Styrofoam plate. A hair clipper (QC5115/15, Philips, The Netherlands) was used to trim hair on the skin sheets to approximately 0.5 mm. Then the skin was cut to a thickness of 1 mm using a Dermatom (GA 630, Aesculap AG & Co. KG, Tuttlingen, Germany) [23]. Circles of 35 cm diameter were cut out with a scalpel. Skin samples were wrapped in aluminium foil and were stored at −30 °C until the day of the experiment.

### 2.3. Incubation of Porcine Ear Skin in a Heatable Diffusion Cell

**Figure 1:**
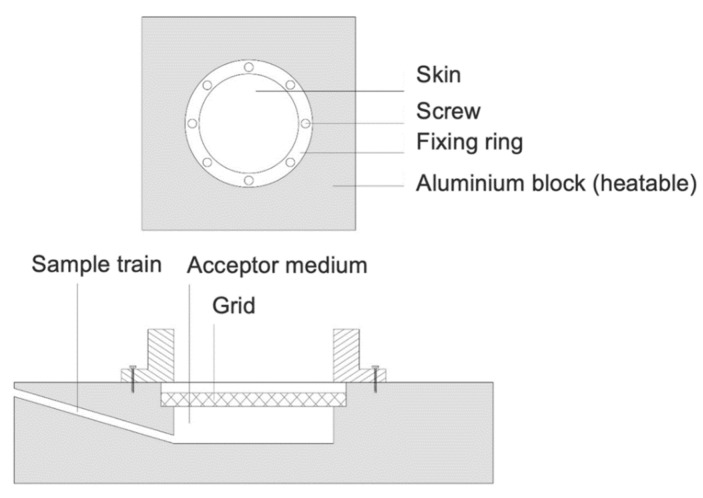
Penetration cell for in-situ CRM measurements.

In order to incubate the porcine ear skin right under the Raman microscope to obtain in situ data, a heatable diffusion cell was developed, and custom made in cooperation with the mechanical workshop of the Institute for Pharmaceutical Sciences, Tuebingen. The incubation cell is inspired by Franz diffusion cells, which are widely used for ex vivo skin penetration studies [24]. A schematic of the cell is shown in Figure 1. It is composed of aluminium and contains an acceptor compartment with a total volume of 7 mL, a sample train, a grid to place the skin on and a donor chamber, which is placed above the skin and held in place by 6 screws. Two sealing rings ensure that the device is tightly sealed. To prevent water loss during incubation time, a piece of parafilm is tightened around the donor compartment and the objective of the Raman Microscope. The incubation cell is heated by two thermocouples with an external controlling device attached, which is set to 32.0 °C throughout the whole incubation time.

For the measurement, the acceptor compartment was filled with 7.0 mL phosphate buffered saline (pH 7.4) and a skin sample was placed on the grid above, which was soaked in PBS. Before applying the sample solution, the skin was tempered for 30 min. After the equilibration time 3.0 mL of the sample solution were applied (infinite dose). Measurements were performed every hour, for a total incubation time of 6 h. All experiments were performed in triplicate on three different days.

### 2.4. Confocal Raman Microscopy (CRM)

For in-situ measuring the incubation cell was placed onto the scan table of an alpha 500 R confocal Raman microscope (WITec GmbH, Ulm, Germany), fixed by 4 pins on the bottom of the cell. The Raman microscope is equipped with a 532-nm excitation laser, a UHTS 300 spectrometer, a DV401-BV CCD detector and a 63× water immersion objective with numerical aperture of 1.0 (W “Plan-Apochromat” 63/1,0 M27, Carl Zeiss, Jena, Germany). To obtain a strong signal without damaging the skin, the laser intensity was set to 25 mW, using a pinhole size of 50 µm. The DV401-BV CCD detector was cooled to −60 °C and a spectral range from 501 cm^−1^ to 1635 cm^−1^ with the spectral centre of 1100 cm^−1^, obtained by an optical grating (1800 g/mm, spectral centre: 1100 cm^−1^). Two-dimensional image scans of 5 µm width and 25 µm in depth were performed, acquiring 10 spectra per line and 50 lines per vertical dimension, with an integration time of 1.5 s per spectra. 

To ensure the suitability of the setup for depth profiling, the depth resolution was measured, by scanning into a silica plate and determining the full width at half maximum of the depth profile corresponding the 521 cm^−1^ band intensity [25]. Also, the thickness of a PET film was measured, in order to test whether valid depth profiles can be obtained.

### 2.5. Data Analysis

**Figure 2:**
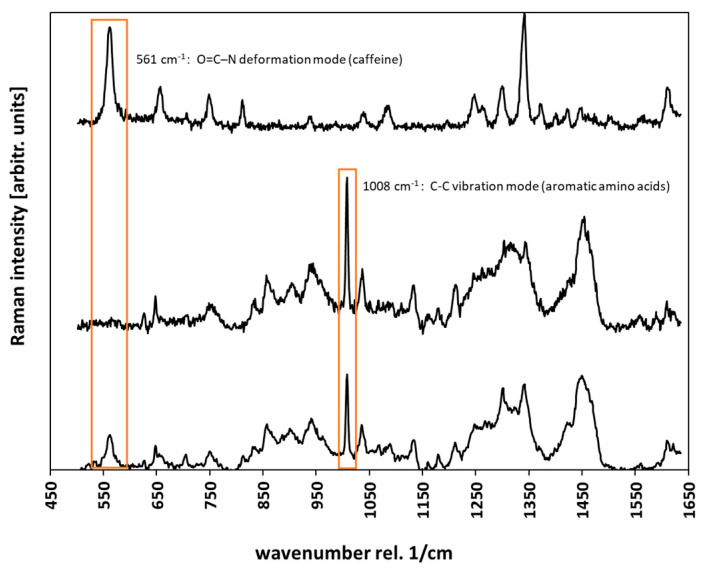
Raman spectra of a 2% caffeine solution (top), stratum corneum (middle) and stratum corneum incubated with caffeine (bottom). The peaks used for calculation of the depth profiles are marked orange.

All recorded spectra were processed by cosmic ray removal and background subtraction using the software Project Plus 4 (WiTec GmbH, Ulm, Germany). Background subtraction was used in the “shape” option (size: 400). For noise reduction principal component analysis (PCA) was performed on all spectra also using the Project Plus 4 software. PCA is an eigenvector-based multivariate statistic method and is commonly used to simplify and structure extensive data sets. The first three principal components were selected for reconstructing a reduced spectrum, containing all essential information, discarding minor variations and noise [26]. Depth penetration profiles of caffeine were determined by calculating the area under the fitted curve (AUC) of the caffeine band at 556 cm^−1^, arising from O=C–N deformation mode. This peak was selected, because it is not interfered with any signals of the skin. The calculation of the AUC was also performed on the Project Plus 4 software using trapezoidal method. The half-maximum of the aromatic amino acid peak at 1008 cm^−1^ was determined, to give the location of the skin surface [27]. All depth profiles were cropped to the skin surface, as the measurement was started around 2 µm above the skin. The arithmetic mean of the aromatic amino acid peak at 1008 cm^−1^ (ring breathing mode) was used to normalize the caffeine signal as it signals attenuation in deeper skin regions and variations over time. Three depth profiles were calculated and extracted out of every image scan, so a total of 9 depth profiles for every solution was used to calculate the mean penetration profile of a specific formulation. To calculate the enhancement ratio, the area under the curve of the depth profiles were determined, using the trapezoidal method and the AUC of the solution with enhancer was divided by the AUC of the solution without enhancer. Representative spectra of the caffeine solution, the stratum corneum and stratum corneum incubated with caffeine are given in Figure 2.

## 3. Results and Discussion

**Figure 3:**
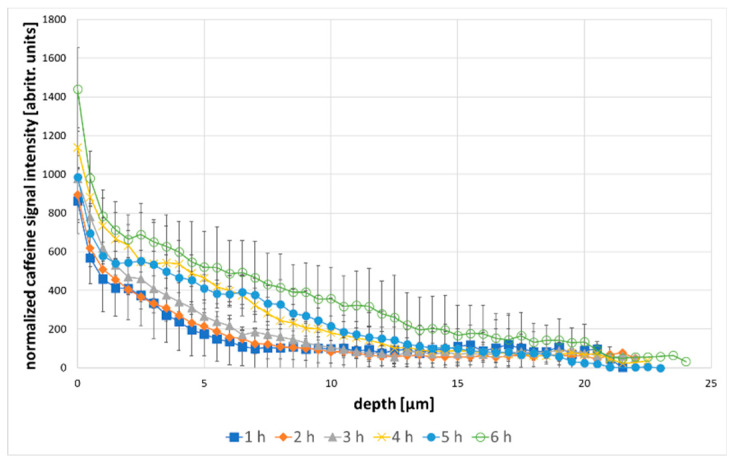
Caffeine depth profiles over 6 h incubation time with 2% caffeine, the error bars are showing the standard deviation.

Following the depth penetration of an API, confocal Raman microscopy has already been performed by several workgroups and has proved promising in replacing destructive methods, such as tape stripping or cryo-segmentation [28,29,30]. It is possible to gain a lot of information in a fraction of the time used for conventional methods. In order to obtain valid data, a suitable microscopic setup is crucial [31]. To validate our setups suitability for depth profiling, the depth resolution was calculated, using the 521 cm^−1^ band of a silica platelet. The depth resolution was found to be 1.86 µm. Furthermore, the thickness of a PET film was measured with the Raman setup and compared to its real thickness of 21.6 µm. The raman results show a thickness of 20.77 µm (standard deviation: ±0.155 µm), which shows an only marginal effect of underestimation of depth, compared to metallurgical objectives [25]. The difference between the real and the measured thickness is therefore 0.83 µm. As the SC is about 15–20 µm thick, which is comparable to the 21.6 µm PET film that was measured, an error of <1 µm across the whole thickness of the SC is deemed acceptable. This shows, that the setup is able to obtain valid depth profiles with a fairly high special resolution, compared to the 5 µm resolution of other instruments [13]. Figure 3 shows the results of in-situ measurements of caffeine penetration during 6 h of incubation with a 2% solution. The Raman intensity of the caffeine band at 556 cm^−1^ is displayed as a function of depth, beginning at the skin surface.

The recorded depth profiles clearly show a steady penetration of caffeine into the skin over the incubation time of 6 h. The presence of caffeine in the SC was already measured after 1 h. For the following 5 h, a steady caffeine penetration was measured. In particular, within the first 10 µm a continuous increase of the caffeine signal over the incubation time was visible. Furthermore, penetration depth increased from 5 µm to 20 µm after 6 h. In fact, the determined penetration profiles coincide with the depth profiles measured by L. Franzen et al. for 12.5 mL/mg caffeine solutions, taking into account that the solutions used in this work are noticeably higher concentrated [32]. While, most of the drug was found in the SC within the first 5 h, which was also found by Laia Rubio et al. [33], after 6 h of incubation, caffein could be found even in 20 µm penetration depth. 

**Figure 4:**
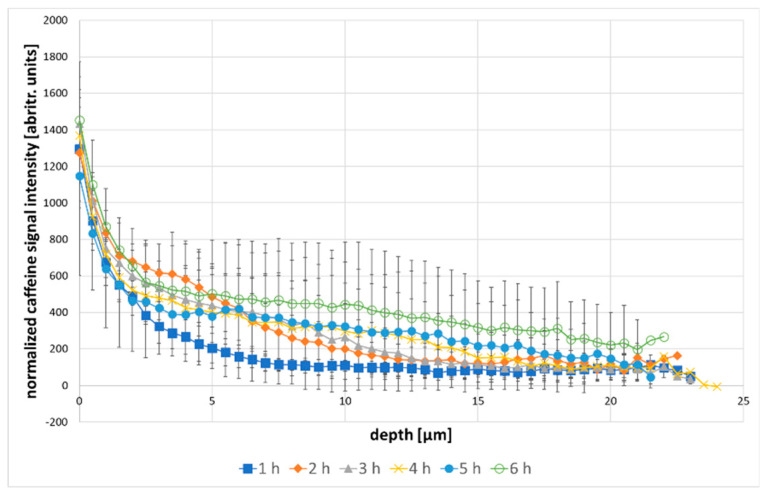
caffeine depth profiles over 6 h incubation time with 2% caffeine +5% 1,2-pentanediol, the error bars are showing the standard deviation.

The effect of 1,2-pentanediol on the caffeine penetration profile was tested to check the effectiveness of the model, in order to establish whether it is possible to detect differences induced by the penetration enhancer. Figure 4 shows the results of in-situ measurements of caffeine penetration during 6 h of incubation with 2% caffeine and 5% 1,2-pentanediol as a penetration enhancer [34].

Over the incubation time of 6 h, a steady increase of caffeine in the skin, as well as an increase in total penetration depth could be detected. Especially between the first and the second hour of incubation, the amount of caffeine in the skin rising was noticeable, while the depth profile between the second and the fifth hour mostly shifted to deeper skin regions. While, the caffeine amount in the upper layers remained the same, the penetration depth increased, which can be deduced by the increasing caffeine signal in deeper skin layers. After 6 h of incubation, caffeine reached a penetration depth of over 20 µm. The amount of caffeine penetrating the skin is generally higher, when adding 1,2-pentanediol to the solution. After 2 h of incubation time, there was already a higher amount of drug in 10 µm depth (normalized caffeine signal: 200 abritr. units) compared to the solution without penetration enhancer (normalized caffeine signal: 82 abritr. units). Also, the penetration depth of caffeine was genuinely higher. After 1 h, the cumulative amount of drug within 10 µm of skin depth (4222 arbitr. units µm). is comparable to the amount of drug after 3 h of incubation time with the formulation without penetration enhancer (4243 arbitr. units µm).

**Figure 5:**
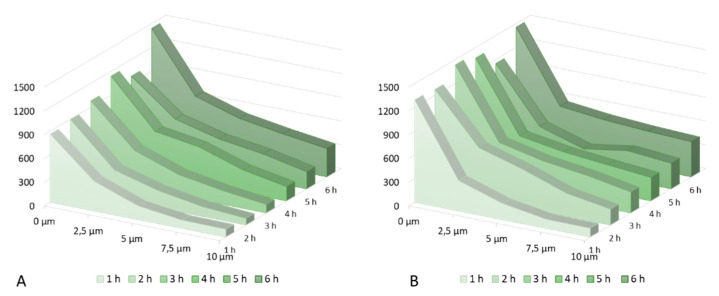
(**A**): Caffeine penetration over 6 h incubation time (2% caffeine solution); (**B**): Caffeine penetration over 6 h incubation time (2% caffeine solution + 1,2-pentanediol).

Figure 5 show the normalized caffeine signal intensity of both solutions tested from the skin surface to 10 µm skin depth in 2.5 µm steps, as most of the differences can be observed within this depth. A shift of the caffeine penetration to deeper skin regions on Figure 5B, caused by the penetration enhancer, was clearly visible. After 2 h the penetration profile of the formulation with enhancer reached almost the same course as the formulation without enhancer after 4 h. Also, the caffeine signal at the skin surface was genuinely higher in the formulation with enhancer, reaching a mean of 1328 arbitrary units µm, in comparison to 1050 arbitrary units µm for the solution without penetration enhancer, as well as the overall amount of drug measured in 10 µm skin depth (273 arbitrary units µm vs. 173 arbitrary units µm). By the end of the incubation time of 6 h, the overall amount of caffeine delivered to the skin was still higher for the formulation containing 1,2-pentanediol, although the difference between both formulations was no long as distinct.

Table 1 shows the calculated enhancement ratios for every time. Enhancement ratios vary between 1.21 and 1.98. It is also shown, that after 2 h, the enhancement effect of 1,2-pentanediol is the clearest. After 3 h the values are decrease again, until the enhancement ratio after 6 h aligns with the ratio after the first hour. This shows that the method can give an insight into penetration kinetics of a topically applied drug.

## 4. Conclusions

In this work, we were demonstrated the effectiveness of a new model for in-situ drug penetration studies. In comparison to conventional methods, such as tape stripping or cryo-segmentation, CRM has the big advantages of being non-destructive and non-invasive. Also, the possibility of in-situ measurements brings further benefits to skin penetration studies, as it is less time consuming. The model we developed successfully shows that it is possible to detect significant differences between two formulations regarding the penetration depth and kinetics of a drug. Understanding the effects of penetration enhancers is important in the development of topically applied formulation, in pharmaceutics, as well as in cosmetics [35]. In particular, in comparative studies between two formulations, the developed method is a promising, time- and resources-saving alternative to the destructive conventional methods. This might be a huge advantage, for example, in the approval of generic formulations. Although, measuring absolute drug concentrations in the skin by CRM is still a challenge, the developed method shows the advantages and the high potential in penetration studies by CRM. 

As this method is very promising, we will continue skin penetration studies to investigate further possibilities and limits. While, we showed the effectiveness of the model for caffeine as a hydrophilic model drug, further drugs, especially lipophilic drugs will be tested. Skin penetration studies of aqueous solutions are possible, as this work shows, so the next steps will be to test the model for in-situ-skin penetration studies with hydrogels and emulsions, as well as with oily solutions.

## Figures and Tables

**Table 1:** cumulative caffeine signal in the skin for every measurement point for the 2% caffeine solution (**A**) and the 2% caffeine solution +5% pentanediol (**B**), and the calculated enhancement ratio.

	AUC _caffeine_ (A) [arbitr.units · µm]	AUC _caffeine_ (B) [arbitr.units · µm]	Enhancement Ratio
**1 h**	3499	4222	**1.21**
**2 h**	3447	6841	**1.98**
**3 h**	4243	6541	**1.54**
**4 h**	5779	6816	**1.18**
**5 h**	5509	6962	**1.26**
**6 h**	7978	9636	**1.21**
